# Scaling of the Berry Phase in the Yang-Lee Edge Singularity

**DOI:** 10.3390/e21090836

**Published:** 2019-08-26

**Authors:** Liang-Jun Zhai, Huai-Yu Wang, Guang-Yao Huang

**Affiliations:** 1 The School of Mathematics and Physics, Jiangsu University of Technology, Changzhou 213001, China; 2 Department of Physics, Tsinghua University, Beijing 100084, China; 3 Institute for Quantum Information & State Key Laboratory of High Performance Computing, College of Computer, National University of Defense Technology, Changsha 410073, China

**Keywords:** Berry phase, Yang-Lee edge singularity, dissipative phase transition, parity-time symmetry-breaking phase transition

## Abstract

We study the scaling behavior of the Berry phase in the Yang-Lee edge singularity (YLES) of the non-Hermitian quantum system. A representative model, the one-dimensional quantum Ising model in an imaginary longitudinal field, is selected. For this model, the dissipative phase transition (DPT), accompanying a parity-time (PT) symmetry-breaking phase transition, occurs when the imaginary field changes through the YLES. We find that the real and imaginary parts of the complex Berry phase show anomalies around the critical points of YLES. In the overlapping critical regions constituted by the (0 + 1)D YLES and (1 + 1)D ferromagnetic-paramagnetic phase transition (FPPT), we find that the real and imaginary parts of the Berry phase can be described by both the (0 + 1)D YLES and (1 + 1)D FPPT scaling theory. Our results demonstrate that the complex Berry phase can be used as a universal order parameter for the description of the critical behavior and the phase transition in the non-Hermitian systems.

## 1. Introduction

Motivated by the pioneering work of Berry [1], the Berry phase in quantum mechanics has become the subject of a variety of theoretical and experimental investigations. It describes a quantum phase effect arising in a cyclic adiabatic process in the parameter space of a quantum system. Since the Berry phase is gauge invariant and geometrical, it is an important and powerful concept in physical science. Application of the Berry phase can be found in broad fields ranging from atomic and molecular to topological materials [2,3,4,5,6,7]. In condensed matters, the recent progress of the Berry phase was the revelation of the close relation between the Berry phase and quantum phase transition (QPT) [8,9,10,11,12,13,14,15,16,17,18,19,20]. Besides QPT and classical phase transition, the dissipative phase transition (DPT) of the Yang-Lee edge singularity (YLES) in the non-Hermitian quantum systems has been studied [21,22,23,24,25,26,27]. Different from QPT, DPT in the non-Hermitian quantum systems is induced by changing the strength of the dissipation. Although the scaling behavior of the Berry phase in QPT has been extensively studied, the scaling behavior of the Berry phase in DPT is still unknown.

When involved with the environment, the system is extended to an open quantum system, which can be characterized by a non-Hermitian Hamiltonian. Some novel physical effects were revealed [28,29,30,31,32]. In the non-Hermitian quantum systems, the Berry phase has been generalized to give a geometrical description of the quantum evolution [33,34,35,36,37,38], and the relationship between the Berry phase and QPT in the non-Hermitian systems has been found [15]. Since the variation caused by DPT can be reflected in the geometry of the Hilbert space, the Berry phase of the non-Hermitian system can capture these changes, as well as the critical behaviors of DPT. Therefore, it is expected that the Berry phase can be exploited as a universal indicator to characterize the scaling behaviors of both QPT and DPT. On the other hand, since DPT is characterized by the spontaneous parity-time (PT) symmetry-breaking phase transition, the non-Hermitian quantum Hamiltonian provides a prototype to study the PT symmetry-breaking phase transition [39,40,41,42,43]. Given the importance of PT symmetric quantum mechanics, it is interesting to study the scaling behaviors of the complex Berry phase in the YLES.

In this paper, we firstly develop a gauge-independent numerical method to calculate the complex Berry phase in the one-dimensional quantum system. This method is applied to a representative example, the one-dimensional quantum Ising model in an imaginary longitudinal field. In this model, an overlapping region exists, which is constituted by the (0 + 1)-dimension ((0 + 1)D) YLES and the (1 + 1)D ferromagnetic-paramagnetic phase transition (FPPT) critical regions. We find that the real and imaginary parts of the Berry phase can be scaled by both the (0 + 1)D YLES and the (1 + 1)D FPPT critical exponents. Our results demonstrate that the complex Berry phase can be the universal indicator of the DPT for both the PT symmetry and PT symmetry-breaking states. The remainder of the paper is organized as follows. In Section 2, the Berry phase of the one-dimensional quantum Ising model in an imaginary longitudinal field is established. Numerical investigation is presented in Section 3. A summary is given in Section 4.

## 2. The Berry Phase of the One-Dimensional Quantum Ising Model in An Imaginary Longitudinal Field

We employ a model of the quantum Ising chain in an imaginary longitudinal field to study the scaling behavior of the Berry phase in the YLES. This model possesses several exotic scaling behaviors in the YLES, such as the divergence of the order parameter, the negative correlation-length exponent in low dimension, and the hybridized Kibble-Zurek scaling (HKZS) mechanism [25,26,27], which can serve as a typical and universal prototype to investigate the scaling behaviors in YLES [23,44,45]. The Hamiltonian reads [44]:

(1) $$\begin{array}{ccc} H & = & -\sum_{n=1}^{L}\sigma_{n}^{z}\sigma_{n+1}^{z}-\lambda \sum_{n=1}^{L}\sigma_{n}^{x}-ih\sum_{n=1}^{L}\sigma_{n}^{z}, \end{array}$$

where $\sigma_{n}^{x}$ and $\sigma_{n}^{z}$ are the Pauli matrices at the *n* site in the *x* and *z* directions, respectively, λ and *h* are the external fields along the transverse and longitudinal directions, and *L* is the lattice size. It is worth stressing that the longitudinal field is imaginary. It has been shown that the imaginary longitudinal field plays the same role as the real field, and as a result, Model (1) has an ordinary FPPT point at $\left(g_{c},h_{c}\right)=\left(0,0\right)$ for L=∞, where $g\equiv \lambda -\lambda_{c}$ and $\lambda_{c}=1$ [46,47]. Besides this FPPT point, there are also critical points for YLES at $\left(g_{\mathrm{YL}}^{L},h_{\mathrm{YL}}^{L}\right)$ for g>0 [44,48], and these YLES points can appear at finite lattice size, while their locations vary with *L* [26]. Accompanied by DPT around the YLES point, the system undergoes a PT symmetry-breaking phase transition. For fixed *g* and $h<h_{\mathrm{YL}}^{L}$ (or $g>g_{\mathrm{YL}}^{L}$ with a fixed *h*), the system is in the PT symmetry phase with real spectra of the ground state. Meanwhile, for $h>h_{\mathrm{YL}}^{L}$ with fixed *g* (or $g<g_{\mathrm{YL}}^{L}$ with a fixed *h*), the system is in the PT symmetry-breaking phase with the ground state energy being complex, and the real part’s energy degenerates with that of the first exited state [23,44,45].

Before going into the details, here, we firstly give a brief remark on the computation of the Berry phase in 1D quantum systems. In the previous studies of the Berry phase in the 1D spin systems, the Hamiltonian was often generalized by applying a rotation to each spin. This rotation spans a 1D parameter space to make the Berry phase well defined [8,9]. Then, by performing a standard procedure based on the Jordan-Wigner transformation and Bogoliubov transformation [49], the generalized Hamiltonian could be diagonalized, and the analytical expression of the Berry phase could be obtained. However, due to the lack of an analytic solution in our model, we have to resort to a numerical calculation. It is checked that if we apply only one type of rotation and directly calculate the Berry phase as Equation (3) (see below), the result will suffer from the gauge problem. To overcome the gauge-dependent nature in Equation (3), we introduce a second parameter through another unequivalent spin rotation. After these manipulations, a gauge-independent formula of the complex Berry phase can be deduced in the 2D parameter space (see Equation (4)).

The transformed Hamiltonian is:

(2) $$\begin{array}{ccc} H & = & U\left(\theta \right)U\left(\eta \right)HU^{+}\left(\eta \right)U^{+}\left(\theta \right), \end{array}$$

where $U\left(\theta \right)=\prod_{n=1}^{L}e^{i\theta \sigma_{n}^{z}/2}$ is the rotation of θ around the *z* direction and $U\left(\eta \right)=\prod_{n=1}^{L}e^{i\eta \sigma_{n}^{x}/2}$ is the rotation of η around the *x* direction. These unitary transformations span a 2D parameter space, which can be applied to the gauge-independent formula of the Berry phase. In addition, the critical behaviors are independent of the parameters θ and η, because the spectrum of the family of Hamiltonians is not affected by the unitary transformations.

The Berry phase for the non-Hermitian systems was firstly studied by Garrison and Wright [33], which is defined as:

(3) $$\begin{array}{c} \beta_{n}=\oint_{c}\langle {\tilde{\Psi }}_{n}\left(\lambda \right)|\nabla_{a}|\Psi_{n}\left(\lambda \right)\rangle d\lambda^{a}, \end{array}$$

where $|\Psi_{n}\left(\lambda \right)\rangle$ and $\langle {\tilde{\Psi }}_{n}\left(\lambda \right)|$ are the normalized right and left eigenvectors of the non-Hermitian Hamiltonian, satisfying $H\left(\lambda \right)|\Psi_{n}\left(\lambda \right)\rangle =E_{n}\left(\lambda \right)|\Psi_{n}\left(\lambda \right)\rangle$, $\langle {\tilde{\Psi }}_{n}\left(\lambda \right)|H\left(\lambda \right)=E_{n}\left(\lambda \right)\langle {\tilde{\Psi }}_{n}\left(\lambda \right)|$, and $\sum_{m}|\Psi_{m}\left(\lambda \right)\rangle \langle {\tilde{\Psi }}_{m}\left(\lambda \right)|=1$. For the non-Hermitian system, the right and left eigenvectors form a biorthonormal basis $\langle {\tilde{\Psi }}_{m}\left(\lambda \right)|\Psi_{n}\left(\lambda \right)\rangle =\delta_{mn}$.

In the 2D parameter space, the complex Berry phase of the ground state of H can be written as:

(4) $$\begin{array}{ccc} \beta_{g} & = & \;\int \int \;\chi (\theta ,\eta )d\theta d\eta \\ & = & i\;\int \int \;d\theta d\eta \sum_{m(\neq g)}\frac{\langle {\tilde{\psi }}_{g}|\partial_{\theta }H|\psi_{m}\rangle \langle {\tilde{\psi }}_{m}|\partial_{\eta }H|\psi_{g}\rangle -\left(\theta ↔\eta \right)}{{\left(E_{g}-E_{m}\right)}^{2}}\\ & = & \beta_{R}+i\beta_{I}, \end{array}$$

where the Berry curvature χ(θ,η) is defined, $\partial_{\theta }=\partial /\partial \theta$, $\partial_{\eta }=\partial /\partial \eta$, $|\psi_{m}\rangle$ is the eigenstate of H, and specifically, $|\psi_{g}\rangle$ is the ground state as indicated by the subscript. $\beta_{R}$ and $\beta_{I}$ are the real and imaginary parts of the Berry phase. Although the expression has the same form as the Berry phase in the Hermitian system, we emphasize that the energy $E_{m}$ is a complex number in general.

## 3. Numerical Results

**Figure 1:**
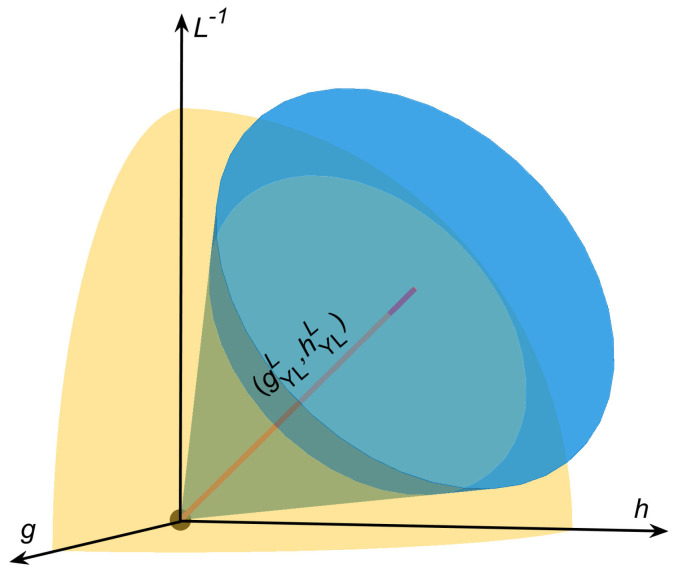
Critical regions near the ferromagnetic-paramagnetic phase transition (FPPT) critical point (origin). The yellow region is the critical region of the FPPT, and the blue cone, which thrusts into the yellow region, is the critical region of the (0 + 1)D Yang-Lee edge singularity (YLES). Critical points ($g_{\mathrm{YL}}^{L}$, $h_{\mathrm{YL}}^{L}$) of the (0 + 1)D YLES link up the red-boldface curve inside the blue cone.

In this section, the static behavior of the Berry phase around the YLES is studied, and the critical region close to the (1 + 1)D FPPT phase transition point is selected. As sketched in Figure 1, the critical region around the (0 + 1)D YLES can appear at finite lattice size, and the (1 + 1)D FPPT critical region appears around the (1 + 1)D critical point with lattice size L=∞. However, at large *L* and small *g*, the critical regions of (0 + 1)D YLES and (1 + 1)D FPPT overlap unavoidably with each other in such a region. According to the HKZS, both the (0 + 1)D YLES and (1 + 1)D FPPT critical theories are applicable simultaneously in this overlapping region, and the coexistence of these two scaling theories can result in a constraint on the scaling functions [27].

**Figure 2:**
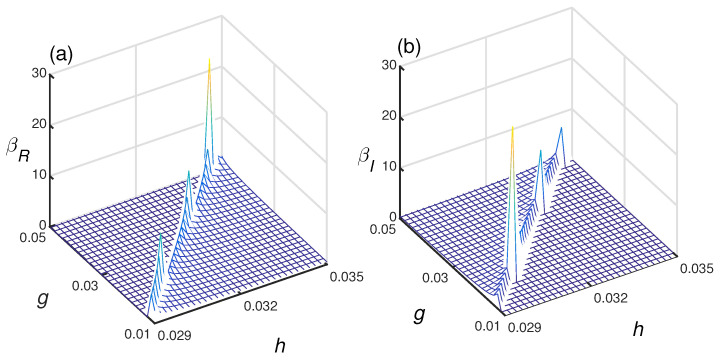
The real part of the complex Berry phase $\beta_{R}$ (**a**) and the imaginary part $\beta_{I}$ (**b**) as a function of Hamiltonian parameters *h* and *g*. Both $\beta_{R}$ and $\beta_{I}$ show anomalies around the YLES points, and the lattice size is L=6.

To demonstrate the relation between the Berry phase and DPT around the YLES, the real and imaginary parts of the Berry phase as a function of the Hamiltonian parameters *g* and *h* are plotted in Figure 2a,b. The lattice size is L=6, and the YLES points lies approximately along the line of $h_{\mathrm{YL}}^{L}=0.14g_{\mathrm{YL}}^{L}+0.027$, which is determined by the order parameters defined as MR=Re[〈ψ˜|M^|ψ〉/〈ψ˜|ψ〉] and MI=Im[〈ψ˜|M^|ψ〉/〈ψ˜|ψ〉] with $\hat{M}=\sum_{n}^{L}\sigma_{n}^{z}/L$ [24,25,26,27]. From Figure 2a,b, we can see that both $\beta_{R}$ and $\beta_{I}$ show anomalies around the YLES points, which demonstrates that the complex Berry phase can detect DPT.

**Figure 3:**
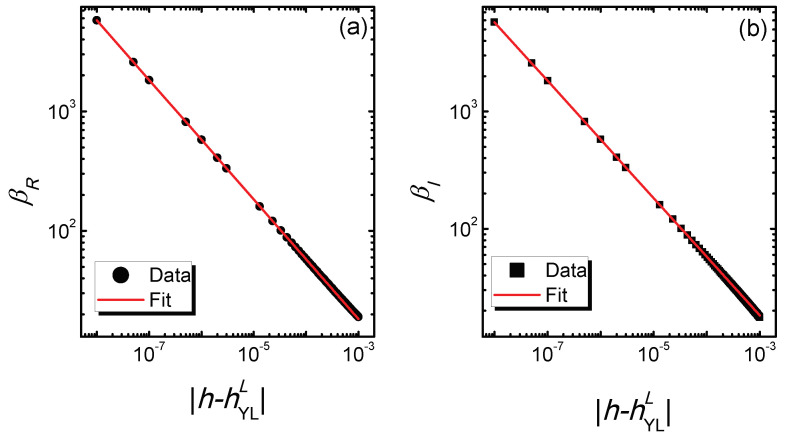
(**a**) The curves of the real part of the complex Berry phase $\beta_{R}$ versus $|h-h_{\mathrm{YL}}^{L}|$ and the fitted line. (**b**) The curves of the imaginary part of the complex Berry phase $\beta_{I}$ versus $|h-h_{\mathrm{YL}}^{L}|$ and the fitted line. The lattice size is L=8, and the YLES point is $\left(g_{\mathrm{YL}}^{L},h_{\mathrm{YL}}^{L}\right)=\left(0.02,0.0180782\right)$. The power law fitting result shows that the exponents are −0.4997 for (a) and −0.5001 for (b).

To further understand the relation between the Berry phase and DPT, we investigate the scaling behaviors of $\beta_{R}$ and $\beta_{I}$. Near the YLES critical points, the numerical study finds that $\beta_{R}$ and $\beta_{I}$ diverge as:

(5) $$\begin{array}{ccc} \beta_{R}\left(h-h_{\mathrm{YL}}^{L}\right) & \propto & |h-h_{\mathrm{YL}}^{L}{|}^{\frac{1}{\delta_{0}}},\\ \beta_{I}\left(h-h_{\mathrm{YL}}^{L}\right) & \propto & |h-h_{\mathrm{YL}}^{L}{|}^{\frac{1}{\delta_{0}}}, \end{array}$$

with $\delta_{0}=-2$ being the value of the (0 + 1)D YLES critical exponent. Figure 3 plots $\beta_{R}$ and $\beta_{I}$ as a function of $|h-h_{\mathrm{YL}}^{L}|$ around $h_{\mathrm{YL}}^{L}$ with L=8. In the double logarithmic coordinates, the curves of $\beta_{R}$ and $\beta_{I}$ versus $|h-h_{\mathrm{YL}}^{L}|$ are straight lines, which indicates that the relation between them satisfies a power law. The fitting results show that the exponents of $\beta_{R}$ and $\beta_{I}$ versus $|h-h_{\mathrm{YL}}^{L}|$ are −0.4997 and −0.5001 respectively, which confirms Equation (5). This result is consistent with the (0 + 1)D YLES scaling theory [24,25], indicating that the scaling behavior of the Berry phase is well described by the (0 + 1)D YLES critical theory. For Figure 3a, $h<h_{\mathrm{YL}}^{L}$, and the Berry Phase is defined in the PT symmetry phase; while for Figure 3b, $h>h_{\mathrm{YL}}^{L}$, and the Berry Phase is defined in the PT symmetry-breaking phase with complex spectra of the ground state. Therefore, this result also demonstrates that the Berry phase could be a universal order parameter in describing both the PT symmetry phase and the PT symmetry-breaking phase. It should be noted that the critical exponent in the scaling functions Equation (5) is the same as that of the scaling functions of order parameters defined in [24,25,26,27], indicating that the scaling behavior of the complex Berry phase is identical to that of $M_{R}$ and $M_{I}$. It could provide a novel way to experimentally detect the Berry phase.

For the (1 + 1)D FPPT, since the imaginary field has the same dimension as the real field, $\beta_{R}$ and $\beta_{I}$ near the FPPT satisfy relations similar to the real longitudinal-field case:

(6) $$\begin{array}{ccc} \beta_{R}\left(g,h,L\right) & = & L^{s}f_{1}\left(gL^{\frac{1}{\nu }},hL^{\frac{\beta \delta }{\nu }}\right),\\ \beta_{I}\left(g,h,L\right) & = & L^{s}f_{2}\left(gL^{\frac{1}{\nu }},hL^{\frac{\beta \delta }{\nu }}\right), \end{array}$$

where β=1/8, δ=15, and ν=1 are the usual critical exponents for the 2D classical Ising universality class, *s* is the (1 + 1)D FPPT critical exponent for the Berry phase, and $f_{1}$ and $f_{2}$ are scaling functions. However, from Equation (5), one finds that $\beta_{R}=\infty$ and $\beta_{I}=\infty$ at the YLES points $\left(g_{\mathrm{YL}}^{L},h_{\mathrm{YL}}^{L}\right)$, due to $\delta_{0}$ being negative. That is, at the YLES points, the scaling functions of Equation (6) become:

(7) $$\begin{array}{ccc} L^{s}f_{1}\left(g_{\mathrm{YL}}^{L}L^{\frac{1}{\nu }},h_{\mathrm{YL}}^{L}L^{\frac{\beta \delta }{\nu }}\right) & = & \infty ,\\ L^{s}f_{2}\left(g_{\mathrm{YL}}^{L}L^{\frac{1}{\nu }},h_{\mathrm{YL}}^{L}L^{\frac{\beta \delta }{\nu }}\right) & = & \infty . \end{array}$$

Since *s* should be a finite constant, one finds that $f_{1,2}\left(g_{\mathrm{YL}}^{L}L^{\frac{1}{\nu }},h_{\mathrm{YL}}^{L}L^{\frac{\beta \delta }{\nu }}\right)=\infty$, which means the variables $g_{\mathrm{YL}}^{L}L^{\frac{1}{\nu }}$ and $h_{\mathrm{YL}}^{L}L^{\frac{\beta \delta }{\nu }}$ should have a relation as:

(8) $$\begin{array}{ccc} g_{\mathrm{YL}}^{L} & = & L^{-\frac{1}{\nu }}f_{3}\left(h_{\mathrm{YL}}^{L}L^{\frac{\beta \delta }{\nu }}\right). \end{array}$$

**Figure 4:**
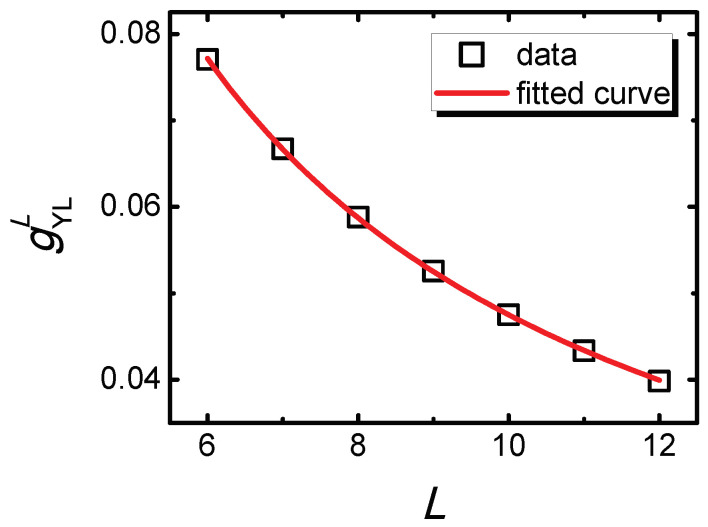
The curve of $g_{\mathrm{YL}}^{L}$ versus *L* and the fitted curve with fixed $h_{\mathrm{YL}}^{L}L^{\frac{\beta \delta }{\nu }}=1.1248$. The fitted curve is $g_{\mathrm{YL}}^{L}=0.4232L^{-0.9499}$.

Equation (8) indicates that $g_{\mathrm{YL}}^{L}$ and $h_{\mathrm{YL}}^{L}$ of YLES points are bound together with an FPPT critical exponent in the overlapping region. Such a relation could be seen as the constraint between the (0 + 1)D YLES and (1 + 1)D FPPT critical theories. To confirm Equation (8) numerically, the curve of $g_{\mathrm{YL}}^{L}$ versus *L* with fixed $h_{\mathrm{YL}}^{L}L^{\frac{\beta \delta }{\nu }}=1.1248$ and the fitted curve are plotted in Figure 4. By a power-law fitting, it is found that the curve satisfies $g_{YL}^{L}\propto L^{-0.9499}$, which agrees with Equation (8).

From Equation (8), one finds that $h_{\mathrm{YL}}^{L}L^{\frac{\beta \delta }{\nu }}$ should be a constant for different lattice size, if $gL^{\frac{1}{\nu }}$ is fixed. Therefore, by fixing $gL^{\frac{1}{\nu }}$ and $\left(h-h_{\mathrm{YL}}^{L}\right)L^{\frac{\beta \delta }{\nu }}$, the scaling function of Equation (6) becomes:

(9) $$\begin{array}{c} \beta_{R,I}\left(L\right)\propto L^{s}. \end{array}$$

**Figure 5:**
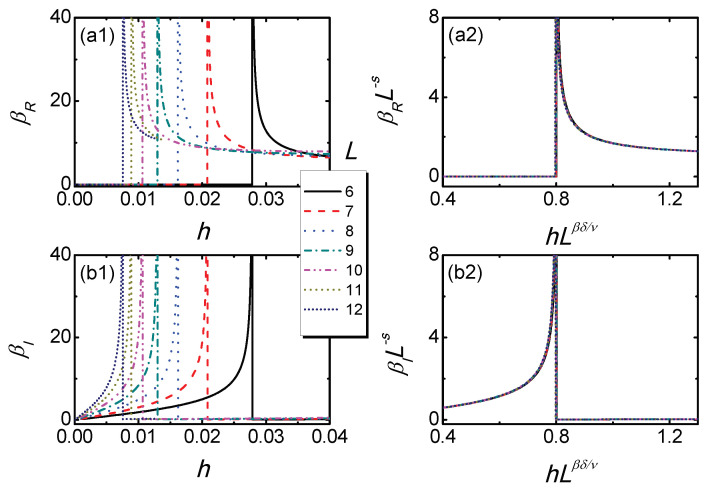
For fixed $gL^{1/\nu }=0.02$, the curves of $\beta_{R}$ versus *h* for different *L* in (**a1**) match with each other in (**a2**) after rescaling according to Equation (6). The corresponding curves for $\beta_{I}$ are shown in (**b1**) and (**b2**).

By a power law fitting, we find that s≃0.8716. We numerically confirm these scaling functions of Equation (6) in Figure 5. In Figure 5a1,b1, $\beta_{R}$ and $\beta_{I}$ versus *h* for different lattice size *L* with $gL^{1/\nu }=0.02$ are plotted. After rescaling by using the (1 + 1)D YLES exponents, the rescaled curves of $\beta_{R}L^{-s}$ and $\beta_{I}L^{-s}$ versus $hL^{\beta \delta /\nu }$ collapse onto each other, as shown in Figure 5a2,b2. These results demonstrate that the behaviors of both the real and imaginary parts of the Berry phase can be well described by the usual FPPT scaling theories.

The numerical results of Figure 3 and Figure 5 confirm that the complex Berry phase could be a good indicator to characterize the scaling behaviors of DPT. Because of the universality of the Berry phase in quantum mechanics, the studies on the scaling behavior of the Berry phase can give knowledge independent of the concrete physical model. It can be easily related to the realistic model by writing the order parameter in terms of the Berry phase, e.g., magnetic moment [8,9], electric polarization [50], or conductance [5].

## 4. Summary

In summary, we developed a gauge-independent Berry phase formula for one-dimensional non-Hermitian quantum systems and applied it to the quantum Ising chain in an imaginary longitudinal field. The scaling behaviors of the complex Berry phase around the YLES of the model were studied. In the overlapping critical regions constituted by the critical regions of the (0 + 1)D YLES and (1 + 1)D FPPT, we showed that both the real and imaginary parts of the Berry phase have anomalies around the YLES points, and their behaviors can be described by both the (0 + 1)D YLES and (1 + 1)D FPPT scaling theories. These results demonstrate that the complex Berry phase could be the universal indicator to detect DPT in the non-Hermitian system. By using the gauge-independent Berry phase formula proposed here, the Berry phase of nonequilibrium states can be calculated, and the complex Berry phase can be applied to study the scaling behaviors of the dynamical phase transition of the non-Hermitian quantum systems. It is also noticed that exotic topological phases were unveiled in the non-Hermitian quantum systems [32], and the Berry phase had a close relation to the topological phase transition [51]. Therefore, the complex Berry phase is expected to characterize the topological phase transition in non-Hermitian systems.

Recently, by measuring the quantum coherence of a probe spin in an Ising bath, the YLES was experimentally found [52,53], and the ground state Berry phase of the Heisenberg XY spin model was also experimentally detected [16]. Therefore, it is expected that the scaling behaviors of the Berry phase of the non-Hermitian systems can be measured, and the results obtained in this paper can be detected therein.

## Abbreviations

The following abbreviations are used in this manuscript:

YLES	Yang-Lee edge singularity
PT	parity-time
FPPT	ferromagnetic-paramagnetic phase transition
QPT	quantum phase transition
DPT	dissipative phase transition
HKZS	Hybridized Kibble-Zurek scaling
